# SNP Arrays

**DOI:** 10.3390/microarrays5040027

**Published:** 2016-10-25

**Authors:** Jari Louhelainen

**Affiliations:** Pharmacy and Biomolecular Sciences, Liverpool John Moores University, James Parsons Building, Byrom Street, Liverpool L3 3AF, UK; j.louhelainen@ljmu.ac.uk; Tel.: +44-151-231-2044

**Keywords:** SNP arrays, human cancers, livestock genetics, animal breeding, array bioinformatics, rare human medical disorders, SNP arrays, human cancers, livestock genetics, animal breeding, array bioinformatics, rare human medical disorders

## Abstract

The papers published in this Special Issue “SNP arrays” (Single Nucleotide Polymorphism Arrays) focus on several perspectives associated with arrays of this type. The range of papers vary from a case report to reviews, thereby targeting wider audiences working in this field. The research focus of SNP arrays is often human cancers but this Issue expands that focus to include areas such as rare conditions, animal breeding and bioinformatics tools. Given the limited scope, the spectrum of papers is nothing short of remarkable and even from a technical point of view these papers will contribute to the field at a general level. Three of the papers published in this Special Issue focus on the use of various SNP array approaches in the analysis of three different cancer types. Two of the papers concentrate on two very different rare conditions, applying the SNP arrays slightly differently. Finally, two other papers evaluate the use of the SNP arrays in the context of genetic analysis of livestock. The findings reported in these papers help to close gaps in the current literature and also to give guidelines for future applications of SNP arrays.

## 1. Introduction

Single Nucleotide Polymorphism (SNP) microarrays are coming to an age as the first papers describing these arrays were published approximately 20 years ago. Since then developments in technology have made screening faster, with more coverage and lower economical cost [1]. SNP microarrays also played a central role when the third-generation human genome map was published with variation data. For single nucleotide mutation screening, these arrays took over from the often cumbersome process of using single strand conformation polymorphism (SSCP) analysis, conformation sensitive gel electrophoresis (CSGE) or denaturing high-performance liquid chromatography (DHPLC). Furthermore, commercial SNP microarrays, such as Affymetrix GeneChip SNP platforms with Copy Number Analyzer for Affymetrix GeneChip (CNAG) algorithms, have been used to study the copy number variation (CNV) frequencies [2]. The findings and methods presented in this collection of papers are important contributions to the use of SNP arrays and provide three valuable perspectives for it: (1) analysis of human cancers; (2) analysis of rare human medical conditions; (3) analysis of livestock with special focus on breeding and bioinformatics. These findings are summarized in this editorial.

## 2. Contributions

Three of the papers published in this Special Issue focus on the use of various SNP array approaches in the analysis of three different cancer types [3,4,5]. Etebari et al. evaluated the role of karyotyping of Non-Hodgkin Lymphomas (NHL) using SNP arrays [3]. Their review highlights genetic alterations of most common subtypes of NHL originating from T and B cells. The aberrations discovered in these studies consist of a spectrum of genes, many of which are linked to human cancers. These include *tumor protein p53* (*TP53*) and *cyclin-dependent kinase inhibitors 2A/2B* (*CDKN2A/CDKN2B)*, which seem to be amongst the most common targets. This review also evaluated the current role of SNP arrays in regards to fluorescent in situ hybridization (FISH), NGS (Next Generation Sequencing) and comparative genomic hybridization (CGH). One of the advantages of SNP arrays is still the cost in comparison to NGS-based approaches. 

The paper by Song et al. summarizes the SNP arrays in regard to hematopoietic malignancies in general, but with emphasis on the clinical variants which are of clinical significance [4]. This review provides a full and detailed update on the genetic alterations of these malignancies. The results are summarized in a table, which quickly gives an overview of the heterogeneity of these diseases. For example, the role of *TP53* in general and the link between *Janus kinase 2* (*JAK2*) V617F mutations in myeloproliferative neoplasms are well known, but the information provided gives an excellent overview of the specifics of expected alterations, suggesting clearly different pathways already from the initiation of these neoplasms. The authors also propose a diagnostic regime, including SNP array screening for patients with hematopoietic malignancies.

Walker et al. provided an update on the current knowledge regarding the role of constitutional CNV in breast cancer [5]. The role of these inherited and de novo CNVs have been studied extensively recently, but one of the points this review raises is the significantly increased size and frequency of CNVs linked to breast cancer. There are also studies reporting rare CNVs overlapping genes involved in breast cancer progression, like *TP53* and *Breast Cancer 1/2* (*BRCA1/BRCA2)*. However, there seems to be a high variation and low reproducibility between studies of these areas. The authors of this review call for more detailed studies of the breakpoints using higher resolution technologies, such as NGS.

High-density SNP analysis combined with Whole Exome Sequencing (WES) can be a powerful tool when looking for individual sequence variants. Nickerson et al. used this approach to analyze patients with a complex neurodegenerative disorder called autosomal recessive cerebellar ataxia (ARCA) [6]. They selected a Maori family whose parents were first cousins. Twelve of the siblings were unaffected but two were diagnosed with the rare form of autosomal recessive spastic ataxia of Charlevoix-Saguenay (ARSACS). For one of the affected individuals the SNP analysis uncovered long continuous stretches of homozygosity (LCSH) and a novel mutation in exon 10 of *SACS* gene was discovered. This gene is on human chromosome 13 and was known as *spastic ataxia of Charlevoix-Saguenay* (*sacsin*); however, currently the approved full name is *sacsin molecular chaperone*. Both siblings were homozygous for this variant, demonstrating the effectiveness of this approach for identifying the underlying genetic phenomena in disorders like this. Another paper in this Special Issue also demonstrated the use of SNP microarrays in analyzing a rare condition, namely split-hand/foot malformation (SHFM) [7]. This condition affects one in 8500–25,000 newborns, with characteristic limb malformations of hands and feet. In some reports mental retardation has also been observed. In many studies duplication of a region on chromosome 10q24 has been linked to this condition. The authors suggest exon 1 of *beta-transducin repeat containing* (*BTRC)* gene (at 10q24.32) to be critical for the split-hand/foot malformation type 3 (SHFM3) phenotype. 

As SNP arrays have become more affordable, a constantly growing amount of data has been generated from species other than human. However, due to variability of typing platforms, allele calling and file formats, the integration of information from various sources has been an obstacle for biological scientists, especially those without advanced bioinformatics skills. In this Special Issue of SNP microarrays, Nicolazzi et al. have concentrated on this problem and present an open source, multi-platform called the SNPConvert suite [8]. Because the source code and graphics user interface (GUI) are freely available, this tool can be upgraded by community-based efforts, hence overcoming the problems usually found with compiled executable files with no active upgrading. They also describe the pitfalls and issues when dealing with quality control of SNP array data from livestock species, especially regarding standardization and integration. The last paper evaluates the use of Lab-on-a-Chip data combined with SNP microarrays in the context of animal breeding and selection [9]. They give examples of SNP loci related to economical traits of common livestock and also suggestions for screening using a combination of Whole Genome Sequencing (WGS) and SNP chips.

## 3. Conclusions

This selection of research papers in this Special Issue focuses on a variety of research areas linked to SNP arrays. These range from bioinformatics of cattle genetics to common human cancer. This Special Issue also gives insight into the diverse use of SNP microarrays. For example, how the SNP arrays can be utilized in tandem with WES, Lab-On-A-Chip and NGS, which are common methods currently used in systematic analysis of genetic alterations in both humans and cattle. Also, as a refined and accurate analysis of the data generated is crucial for the outcome, within this Issue, guidelines are provided for bioinformatics associated with these processes, together with open source analysis tools. In summary, the collection of these seven papers emphasizes the importance of understanding various aspects in the use of SNP arrays, with examples from a range of mammalian applications. These reports should also prove to be an asset for anyone intending to use SNP microarrays in the future.
